# A Case Report of Reversible Mitochondrial Bioenergetic Dysfunction in PBMCs in Anti-GAD65–Associated Cerebellar Ataxia

**DOI:** 10.1007/s12311-026-02025-y

**Published:** 2026-05-20

**Authors:** Natália Huňarová, Andrea Ižarik Verešpejová, Milan Grófik, Štefan Sivák, Egon Kurča, Martin Kolísek, Pavol Skáčik

**Affiliations:** 1 Institute of Medical Biochemistry, Jessenius Faculty of Medicine in Martin, Comenius University in Bratislava, Martin, Slovakia; 2 Biomedical Centre Martin, Jessenius Faculty of Medicine in Martin, Comenius University in Bratislava, Martin, Slovakia; 3 Clinic of Neurology, Jessenius Faculty of Medicine in Martin, University Hospital Martin, Comenius University in Bratislava, Kollarova 2, Martin, 036 01 Slovakia

**Keywords:** Anti-GAD ataxia, Cerebellar ataxia, Mitochondrial dysfunction, Peripheral blood mononuclear cells, Immunometabolism

## Abstract

Immune-mediated cerebellar ataxias lack dynamic biomarkers that reflect systemic metabolic changes during treatment. In this longitudinal case of anti-GAD65 cerebellar ataxia, high-resolution respirometry of peripheral blood mononuclear cells (PBMCs) revealed treatment-associated shifts in mitochondrial bioenergetic organization that occurred in temporal association with clinical improvement. A 61-year-old woman presented with a 6-month history of progressive gait instability, downbeat nystagmus, dysarthria. Diagnostic workup revealed a highly elevated anti-GAD65 antibody index (> 400.0), while brain MRI and routine cerebrospinal fluid analysis were unremarkable, confirming a diagnosis of anti-GAD-associated cerebellar ataxia. Serial high-resolution respirometry was used to assess PBMC mitochondrial function at baseline and following successive therapies. At baseline, the patient exhibited a Complex I–dominant profile with suppressed Complex II activity, reduced glycerophosphate dehydrogenase (GpDH) flux, and impaired mitochondrial coupling. While corticosteroids yielded negligible metabolic or clinical changes, subsequent plasmapheresis was followed by increased Complex II and GpDH flux, normalized coupling parameters, and expanded respiratory reserve. These bioenergetic shifts paralleled significant clinical recovery, as evidenced by a reduction in the Scale for the Assessment and Rating of Ataxia (SARA) score from 17 to 12, and were further accompanied by a decrease in the anti-GAD65 antibody index. This case suggests that PBMC mitochondrial bioenergetics may reflect systemic metabolic changes during treatment in anti-GAD ataxia. These observations provide a preliminary metabolic perspective on immunotherapy response and warrant validation in larger patient cohorts.

**Supplementary Information:**

The online version contains supplementary material available at 10.1007/s12311-026-02025-y.

## Introduction

Anti–glutamic acid decarboxylase (anti-GAD)–associated cerebellar ataxia is a rare immune-mediated neurological disorder characterized by a subacute or chronic progressive ataxic syndrome, commonly presenting with downbeat nystagmus, dysarthria, and gait instability. The condition predominantly affects women in their sixth decade of life and may follow either a subacute or insidious chronic course. High anti-GAD65 antibody titers in serum or cerebrospinal fluid represent a diagnostic hallmark [1].

Anti-GAD–associated ataxia frequently occurs in the context of systemic autoimmunity, with a substantial proportion of patients exhibiting coexisting autoimmune disorders, including type 1 diabetes mellitus, autoimmune thyroid disease, and pernicious anemia. In addition to cerebellar dysfunction, extracerebellar manifestations may be present, such as epilepsy, ophthalmoplegia, and features within the stiff-person spectrum, reflecting a broader involvement of the central nervous system [2, 3].

Emerging evidence in immunometabolism suggests that mitochondrial bioenergetics govern immune cell activation and systemic inflammatory responses [4]. In various autoimmune pathologies, activated immune cells exhibit a shift toward a “metabolically rigid” phenotype, characterized by Complex I dominance and reduced flexibility in alternative electron-entry pathways [5, 6]. Such bioenergetic profiles may serve as functional indicators of systemic immune dysregulation; however, this metabolic signature remains unexplored in the context of anti-GAD–associated disorders.

In this report, we describe a case of anti-GAD cerebellar ataxia in which longitudinal high-resolution respirometry of peripheral blood mononuclear cells (PBMCs) was used to track the disease course. Our findings suggest dynamic mitochondrial bioenergetic alterations during the disease course and treatment, which occurred alongside clinical evolution, indicating that PBMC bioenergetics may represent a potential non-invasive biomarker for monitoring immunotherapy efficacy in immune-mediated ataxia.

## Case Presentation

### Patient Information

A 61-year-old Caucasian woman with a history of arterial hypertension and autoimmune hypothyroidism, treated with levothyroxine, presented with a history of progressively worsening dizziness, gait instability, visual disturbances (including diplopia and blurred vision), and speech impairment. She initially experienced an acute episode of vertigo in April 2024 that resolved spontaneously, followed by a second episode in November 2024. After the second episode, her symptoms—including imbalance, dysarthria, and visual disturbances (namely diplopia and blurred vision)—progressively deteriorated. There was no family history of neurological disorders, and she had no prior exposure to neurotoxic agents. The interval between progressive symptom onset and the establishment of a definitive diagnosis with initiation of targeted therapy was approximately 10 months.

### Clinical Findings

Neurological examination revealed a cerebellar syndrome with prominent ocular motor dysfunction, including downbeat nystagmus, saccadic oscillations, and impaired smooth pursuit and saccadic eye movements. Speech assessment demonstrated cerebellar dysarthria. The clinical picture was dominated by an axial cerebellar syndrome, characterized by marked truncal ataxia and impaired stance and gait, with severe difficulty performing tandem gait; gait instability further worsened in the absence of visual input. Appendicular ataxia was also present, with mild dysmetria affecting both the upper and lower limbs. Vibration sense, assessed using a tuning fork, was preserved (8/8). Muscle tone was normal, and no features of parkinsonism were observed. Deep tendon reflexes were brisk, with bilateral extensor plantar responses. There were no signs of autonomic dysfunction, including urinary incontinence or orthostatic hypotension.Neuropsychological evaluation demonstrated mild executive and visuospatial dysfunction accompanied by depressive symptoms. The initial Scale for the Assessment and Rating of Ataxia (SARA) score was 17, consistent with moderate-to-severe functional impairment (see timeline in Table 1).

**Table 1:** Timeline of clinical course, therapeutic interventions, and PBMC respirometry sampling in relation to treatment. PBMC samples were collected at baseline (pre-treatment) and within 24 h after each intervention (corticosteroid therapy and final TPE session). Clinical follow-up was performed within 48 h after completion of TPE

Time point	Clinical event / Findings	Intervention	PBMC respirometry
April 2024	Initial onset: acute dizziness; spontaneous resolution	-	-
November 2024	Second episode of vertigo followed by chronic progressive cerebellar syndrome (gait instability, dysarthria, oscillopsia) over ~ 10 months	-	-
~ 10 months from onset (Initial assessment & hospitalization)	Established cerebellar syndrome; SARA 17; serum anti-GAD65 > 400 (index)	-	Baseline sample (pre-treatment)
Corticosteroid therapy	No clinical improvement	IV methylprednisolone followed by oral prednisone	Second sample (within 24 h after corticosteroid therapy)
Plasmapheresis (TPE)	Persistent symptoms prior to escalation	5 sessions of TPE	Third sample (within 24 h after final TPE session)
Follow-up (< 48 h after TPE)	Clinical improvement: SARA 12, serum anti-GAD65 325.0 (index)	-	-

### Diagnostic Assessment

A thorough evaluation for the etiology of ataxia was performed, including screening for infectious, metabolic, autoimmune, paraneoplastic, genetic, and neurodegenerative causes (Supplementary Table S1 and S2). Anti-GAD65 antibodies were quantified using ELISA and expressed as an index relative to the assay reference standard, revealing a markedly elevated value (> 400.0; reference range 0.00–1.00). Borderline antinuclear antibody (ANA; HEp-2) positivity was detected; however, extended autoimmune serological testing, as well as evaluation for endocrine disorders (including diabetes mellitus) and nutritional deficiencies, revealed no clinically significant abnormalities. Comprehensive paraneoplastic screening, including onconeural antibody testing and computed tomography of the thorax, abdomen, and pelvis, showed no evidence of malignancy.

Cerebrospinal fluid (CSF) analysis, including routine cytology and biochemical assessment, was unremarkable (Supplementary Table S1). Neuroimaging with brain and spinal cord magnetic resonance imaging (MRI) demonstrated stable chronic microvascular changes (Fazekas grade 2) and a pre-existing arachnoid cyst, without evidence of cerebellar or spinal atrophy. Electrophysiological studies excluded peripheral neuropathy. Somatosensory evoked potentials following bilateral stimulation of the median and tibial nerves showed normal peripheral and cortical response latencies, preserved amplitudes, and intact central conduction times. Visual evoked potentials (VEPs) performed at diagnosis demonstrated bilaterally prolonged P100 latencies with preserved amplitudes (left: 123 ms, 7.2 µV; right: 123 ms, 6.7 µV).

Videonystagmography (VNG) revealed abnormalities of fixation, characterized by downbeat nystagmus with superimposed saccadic oscillations (square-wave jerks), severe impairment of smooth pursuit, and saccadic dysmetria, more pronounced in the vertical than in the horizontal plane. Video head impulse testing (vHIT) demonstrated preserved function of all six semicircular canals, with normal gain values and no covert or overt corrective saccades. Genetic testing for ataxia-related disorders was unremarkable (Supplementary Table S2). Based on these findings, together with the systematic exclusion of alternative neuroinflammatory, degenerative, and genetic etiologies, a diagnosis of anti–GAD-associated cerebellar ataxia was established.

### Therapeutic Interventions

Initial management targeted acute neuroinflammation with high-dose pulse corticosteroid therapy, comprising 3 g of intravenous methylprednisolone followed by an oral prednisone taper (60 mg/day). Despite this regimen, the patient remained clinically refractory, showing no objective improvement in ataxic symptoms or functional disability.

Given the lack of response to corticosteroids and the progressive disease course, therapeutic plasma exchange (TPE) was initiated as a second-line intervention. The patient completed five sessions of high-volume plasmapheresis without adverse events. Following the final TPE session, a clear clinical inflection point was observed. The patient reported a reduction in vertigo and dizziness, accompanied by objective improvement in cerebellar function, including both axial and appendicular ataxia, as evidenced by a 5-point reduction in the Scale for the Assessment and Rating of Ataxia (SARA) score (from 17 to 12). A slight improvement in dysarthria was also noted, whereas oculomotor abnormalities remained unchanged.

The serum anti–GAD65 antibody index decreased to 325.0 (reference range 0.00–1.00), as measured by ELISA. Follow-up visual evoked potentials obtained after initiation of immunotherapy demonstrated mildly prolonged P100 latencies bilaterally (left: 115 ms, amplitude 7.4 µV; right: 116 ms, amplitude 8.2 µV), consistent with mild demyelinating involvement of the visual pathways and showing improvement compared with prior assessment. These clinical and electrophysiological improvements were paralleled by recovery of mitochondrial bioenergetic parameters.

### PBMC Mitochondrial Bioenergetics Profiling

To investigate systemic metabolic correlates of treatment response, we performed longitudinal high-resolution respirometry (Oroboros O2k) on peripheral blood mononuclear cells (PBMCs) at three key time points: baseline, post-corticosteroid therapy, and post-plasmapheresis. Mitochondrial function was assessed using standardized coupling control (CCP) and substrate–uncoupler–inhibitor titration (SUIT) protocols in both intact and permeabilized cells (Supplementary Material, Fig. 1).

**Fig. 1:**
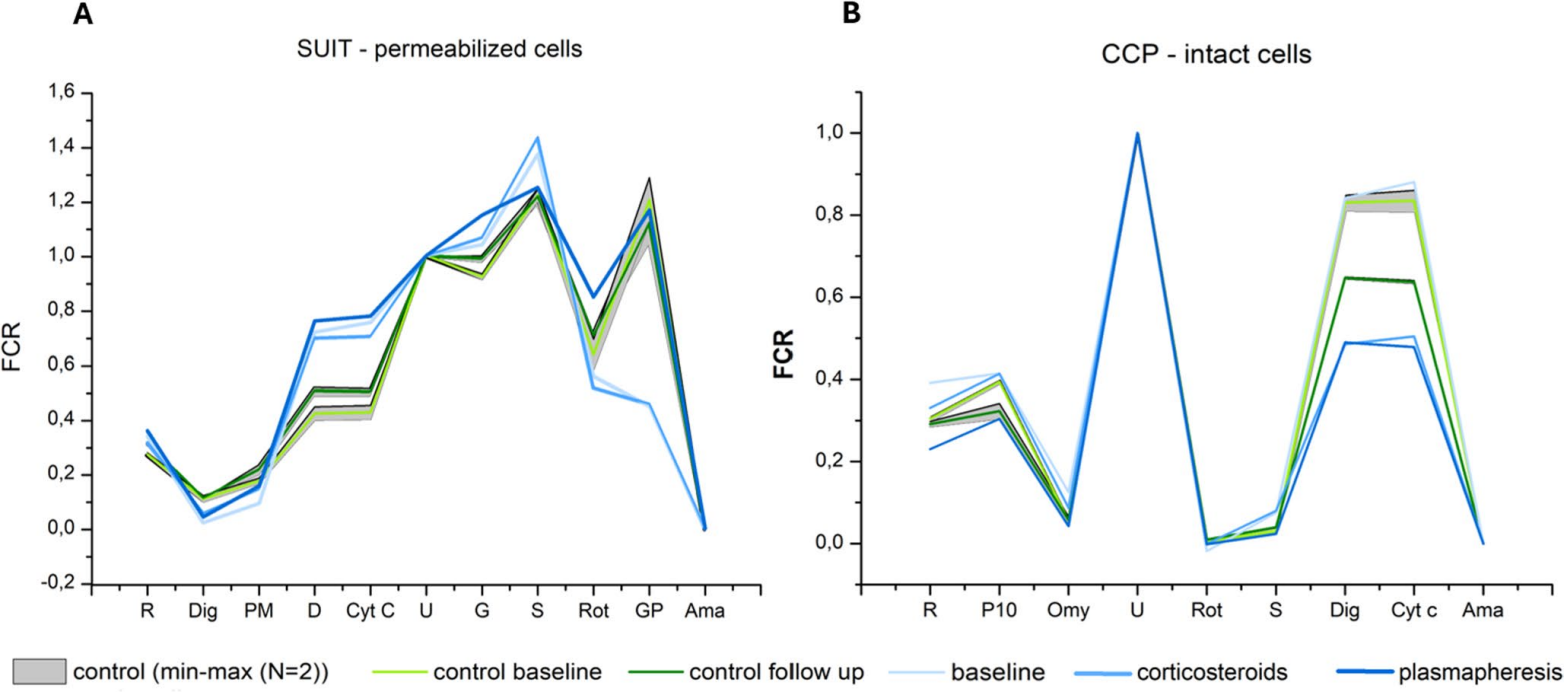
Mitochondrial respiration profiles in PBMCs (**1A**) SUIT protocol in permeabilized PBMCs illustrating the sequential engagement of Complex I, Complex II, and glycerol-3-phosphate–dependent respiration. Control samples were measured in duplicate (*N* = 2); the grey shaded area represents the min–max range, and the central line indicates the mean. Patient samples were measured once per protocol. (**1B**) High-resolution respirometry profiles in intact PBMCs obtained using the coupling control protocol (CCP), showing routine respiration (R), oligomycin-induced leak (Omy), maximal electron transport system capacity (U), and residual oxygen consumption across treatment stages

Respiratory measurements were evaluated using both absolute oxygen flux and normalized bioenergetic indices describing mitochondrial coupling and pathway contributions. These included the respiratory control ratio (RCR), leak control ratio (L/R), and flux control ratios reflecting the relative contribution of individual respiratory pathways (Fig. 1).

#### Methodological Considerations

Because the study was conducted in a clinical setting with repeated sampling during treatment, the amount of biological material available for analysis was limited. Consequently, intact-cell and permeabilized-cell protocols were performed in parallel chambers rather than repeated technical replicates. Given the exploratory nature of a single-case observation and comparison with a single age-matched control, the bioenergetic data are presented as descriptive measurements rather than population reference values.

Leak respiration was assessed in a context-specific manner: as NADH-linked proton leak in permeabilized cells and as oligomycin-inhibited leak in intact cells. These complementary measures provided a comprehensive evaluation of mitochondrial membrane integrity and coupling efficiency.

### Baseline Mitochondrial Function

**Fig. 2:**
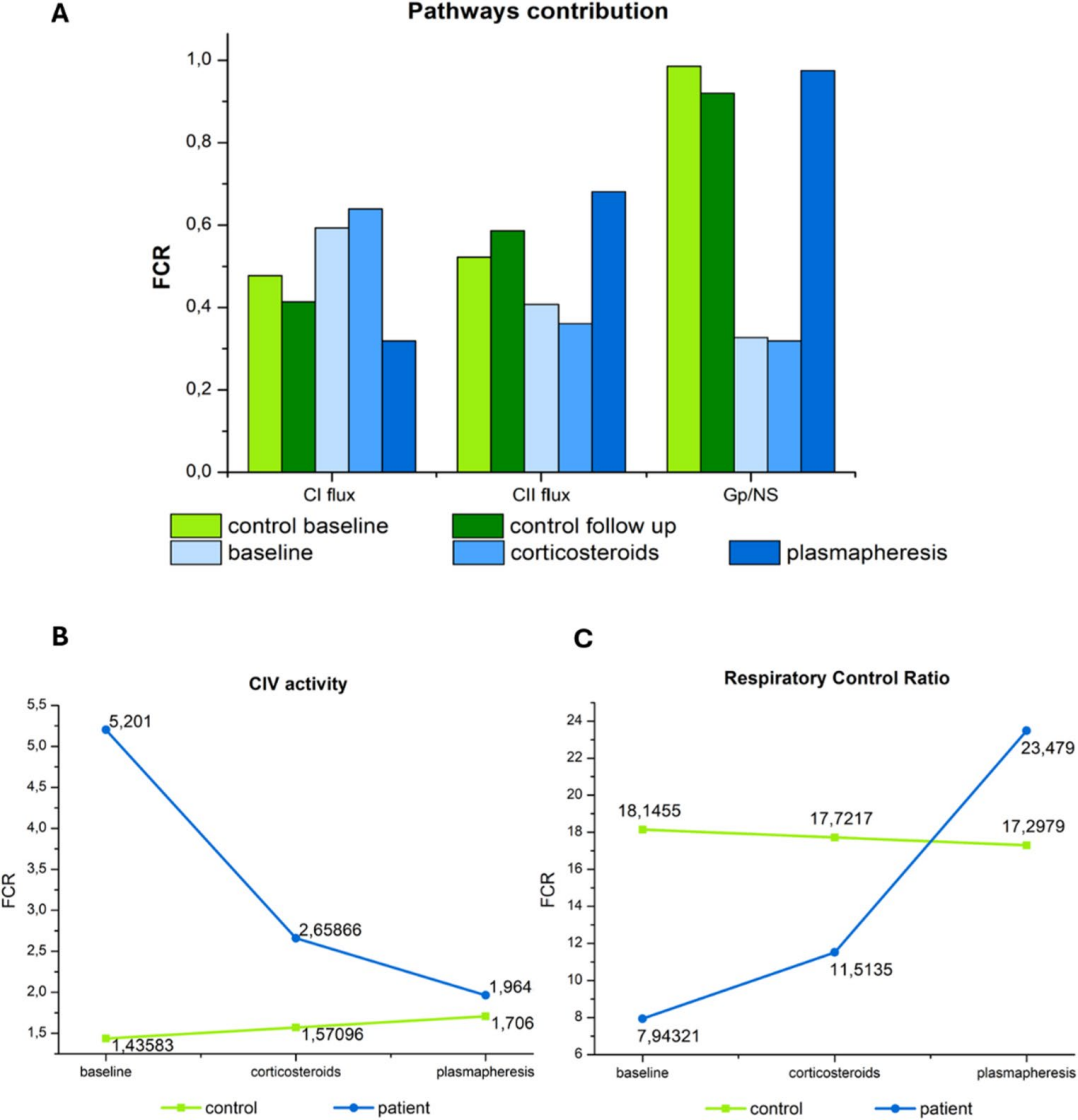
Redistribution of respiratory pathway contributions, normalization of Complex IV activity and respiratory control ratio following plasmapheresis. (**2A**) Relative contribution of CI, CII, and glycerol-3-phosphate–linked respiration normalized to NS pathway capacity across treatment stages. Baseline and corticosteroid-treated samples demonstrated CI-dominant respiration with suppression of alternative electron-entry pathways. After plasmapheresis, CI contribution decreased while CII and GpDH contributions increased markedly, indicating restored metabolic flexibility. (**2B**) CIV activity across treatment stages, demonstrating elevated activity at baseline with progressive normalization following immunomodulatory therapy. (**2C**) RCR across treatment stages in the healthy control, baseline sample, post-corticosteroid sample, and post-plasmapheresis sample. Baseline PBMCs exhibited markedly reduced RCR, indicating impaired coupling efficiency. Plasmapheresis resulted in a substantial increase in RCR beyond control levels. Together, these findings indicate a reversible, functional mitochondrial alteration rather than irreversible structural damage

**Fig. 3:**
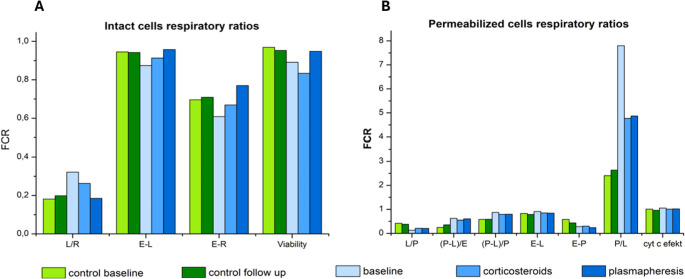
Respiratory coupling and reserve indices in intact and permeabilized PBMCs. (**3A**) Respiratory ratios derived from intact PBMCs, including leak-to-routine ratio (L/R), coupling efficiency (E–L), respiratory reserve (E–R), and cell viability. (**3B**) Respiratory ratios derived from permeabilized PBMCs, including leak control, phosphorylation control, electron transfer efficiency, and cytochrome c effect. Baseline measurements showed impaired coupling and reduced reserve capacity. Corticosteroid therapy induced only minor changes. In contrast, plasmapheresis was associated with improved coupling efficiency, increased respiratory reserve, and preserved membrane integrity in both intact and permeabilized cells

At baseline, the patient’s PBMCs exhibited a distinct metabolic signature of immune stress, characterized by a pronounced Complex I (CI)–dominant respiratory pattern accompanied by reduced Complex II (CII) and glycerol-3-phosphate dehydrogenase (GpDH) activities (Fig. 2A). This profile was associated with altered coupling parameters (Figs. 2B and 3) and a reduced respiratory control ratio (RCR; Fig. 2C) compared with healthy controls, indicating bioenergetic constraint prior to intervention.

### Longitudinal Outcomes and Metabolic Recovery

Clinical and metabolic responses were closely aligned throughout the therapeutic course. Following corticosteroid therapy, the patient showed no clinical improvement and remained symptomatic; respirometry reflected this lack of clinical response, demonstrating only minimal bioenergetic changes (Figs. 2 and 3).

In contrast, plasmapheresis was associated with a marked metabolic shift. High-resolution respirometry demonstrated restoration of CII-supported respiration and GpDH flux (Fig. 1A), a shift of coupling parameters toward values observed in the control sample, and an expansion of respiratory reserve (Fig. 3). The respiratory control ratio (RCR) increased substantially, from 7.9 at baseline to 23.5 post-plasmapheresis, exceeding values observed in healthy controls (Fig. 2C). These metabolic changes occurred in temporal association with clinical improvement, reflected by a 5-point reduction in the SARA score (from 17 to 12) and a decrease in the anti-GAD65 antibody index (additional data are provided in Supplementary Tables S3–S6).

## Discussion

Anti–GAD-associated ataxia represents an important cause of late-onset cerebellar ataxia and should be considered in the differential diagnosis of progressive adult-onset ataxia [7]. The disorder is often associated with an unfavorable prognosis, particularly when immunotherapy is initiated late, frequently resulting in persistent cerebellar deficits and long-term disability [2, 3]. In the present case, symptom onset occurred in the sixth decade in the context of concomitant autoimmune thyroid disease, supporting an autoimmune etiology, while extensive diagnostic evaluation excluded alternative causes of ataxia.

Retrospectively, it was not possible to determine whether the initial episodic vestibular symptoms were directly related to anti–GAD-associated ataxia; however, early episodic manifestations have been reported [8]. The presence of pyramidal signs necessitates consideration of alternative or overlapping diagnoses, particularly multiple system atrophy of the cerebellar type (MSA-C) [9]. Although our patient did not meet diagnostic criteria for MSA-C, future phenotypic evolution cannot be entirely excluded [10], and systematic assessment for parkinsonism and autonomic dysfunction remains essential. Additionally, abnormalities in muscle tone should be evaluated given the overlap with stiff-person spectrum disorders [11].

Complementary diagnostic approaches, including assessment of peripheral vestibular function and the detection of subclinical involvement using evoked potentials, may further aid in differentiating causes of late-onset ataxia [12, 13]. In our case, visual evoked potentials demonstrated bilaterally prolonged P100 latencies with preserved amplitudes. Although rarely reported, optic pathway involvement has been suggested in anti–GAD-associated disorders, supporting a multifocal pattern of central nervous system involvement [14]. Potential alternative causes of VEP latency prolongation include ophthalmologic factors; however, the presence of bilateral pseudophakia in this patient is unlikely to account for the observed delay. Ocular motor disturbances, such as nystagmus or saccadic intrusions, may also influence VEP recordings, although these typically affect amplitudes rather than latencies, and amplitudes were preserved in this case [15]. Partial improvement on follow-up VEPs after immunotherapy should be interpreted cautiously given the single-case nature of this observation.

Late-onset cerebellar ataxia requires a comprehensive diagnostic approach. In our patient, elevated serum anti-GAD65 antibodies in the context of autoimmune thyroid disease supported an autoimmune etiology. CSF analysis was unremarkable, with normal protein levels, no pleocytosis, and absence of oligoclonal bands; CSF anti-GAD antibodies were not assessed. Notably, oligoclonal bands are present in approximately 60–70% of anti-GAD ataxia cases, and their absence does not exclude the diagnosis [8].

The patient demonstrated only partial clinical improvement, primarily in axial symptoms, with only mild improvement in speech and appendicular ataxia, while ocular motor abnormalities remained unchanged. This pattern is consistent with the limited therapeutic responsiveness of anti–GAD-associated ataxia in more advanced stages and may reflect impaired cerebellar compensatory capacity [2].

Beyond diagnostic evaluation, we explored potential pathophysiological mechanisms by assessing treatment-dependent mitochondrial abnormalities in PBMCs. At baseline, PBMCs exhibited a bioenergetic phenotype characterized by relative CI-dominant respiration, reduced engagement of CII and GpDH-linked pathways, impaired coupling, and a decreased respiratory control ratio. Additionally, elevated Complex IV activity may reflect a compensatory response to inefficient electron transport or increased CI flux. This pattern may be compatible with reduced metabolic flexibility associated with chronic immune activation and mitochondrial stress [16, 17].

Activated immune cells undergo profound metabolic reprogramming to sustain cytokine production, proliferation, and inflammatory signaling. Increased reliance on NADH-linked electron entry through Complex I, together with reduced engagement of alternative pathways such as Complex II and GpDH, may indicate reduced metabolic flexibility and preferential routing of electrons through pro-inflammatory bioenergetic states. Reduced respiratory control ratio and increased leak respiration further support mitochondrial stress and inefficient oxidative phosphorylation, features commonly associated with inflammatory immune-cell activation [4, 16, 17]. Similar immunometabolic remodeling has been described across multiple immune-mediated disorders, including multiple sclerosis, rheumatoid arthritis, systemic lupus erythematosus, and inflammatory bowel disease, where activated PBMCs exhibit increased glycolytic dependence, altered mitochondrial coupling, and redistribution of electron-entry pathways [4, 5].

The absence of clinical response to corticosteroid therapy was paralleled by minimal bioenergetic change, with persistent imbalance between CI-driven respiration and alternative electron-entry pathways. Although glucocorticoids can modulate immune metabolism by suppressing aerobic glycolysis and promoting alternative substrate utilization [18], this effect appeared insufficient to substantially modify the observed bioenergetic profile in this case.

In contrast, plasmapheresis was associated with a shift toward a bioenergetic profile more similar to that observed in the control sample, including restoration of CII and GpDH activity, normalization of coupling parameters, and substantial improvement in respiratory control ratio. Changes in Complex IV activity may reflect rebalancing of electron transport chain activity, although oxidative stress was not directly measured in this study. These systemic bioenergetic changes paralleled the clinical course, with reduction in subjective symptoms and improvement in cerebellar syndrome, as reflected by a 5-point decrease in the SARA score.

The concordance between clinical evolution and mitochondrial dynamics suggests that PBMC bioenergetic profiling may reflect systemic immunometabolic processes occurring during autoimmune ataxias. To our knowledge, this is the first report describing treatment-associated changes in PBMC mitochondrial function in anti-GAD–associated ataxia. During the active disease phase, PBMCs exhibited a bioenergetic signature consistent with immune stress, which shifted toward a profile more similar to that observed in the control sample following clinical improvement.

From a translational perspective, PBMC respirometry provides a functional assessment of mitochondrial activity in accessible immune cells, complementing conventional biomarkers such as antibody titers. If validated in larger cohorts, such bioenergetic profiling may help identify patterns of systemic metabolic adaptation during immunotherapy [19].

However, as a single-case observation, these findings must be interpreted with caution. Further longitudinal studies are needed to determine the clinical utility of PBMC bioenergetics as a biomarker and to clarify its relationship to disease activity, long-term outcomes, and the underlying mechanisms of anti-GAD–mediated neuroinflammation.

## Conclusion

This case suggests that PBMC mitochondrial bioenergetic alterations observed in this case appeared dynamic and potentially reversible and paralleled the clinical disease course. While corticosteroid therapy was not associated with clear clinical or bioenergetic changes, plasmapheresis was followed by improvement in both neurological function and mitochondrial respiratory parameters. Although limited to a single observation, these findings raise the possibility that PBMC bioenergetic profiling may represent a potential peripheral indicator of immunometabolic activity during treatment in immune-mediated cerebellar disorders, warranting further investigation in larger studies.

## Supplementary Information

Below is the link to the electronic supplementary material.

Supplementary Material 1

Supplementary Material 2

## Data Availability

Data are available from the corresponding author upon reasonable request.

## Declarations

### Declarations of AI Use

The authors utilized ChatGPT 5.2 (OpenAI, GPT-based large language model) and Google built-in AI tool solely for language editing to improve grammar and stylistic fluency. The final scientific content, data interpretation, and conclusions remain the sole responsibility of the authors.

### Clinical Trial Registration

Clinical trial number: not applicable.

## Abbreviations

ANA: antinuclear antibodies
ANCA: antineutrophil cytoplasmic antibodies
Ama: antimycin A
AsTm: ascorbate + N, N,N′,N′-tetramethyl-p-phenylenediamine
AzD: azide
CCP: coupling control protocol
CCCP: carbonyl cyanide-m-chlorophenylhydrazone
CI: mitochondrial Complex I
CII: mitochondrial Complex II
CIV: mitochondrial Complex IV
CMV: cytomegalovirus
CSF: cerebrospinal fluid
Cyt c: cytochrome c
Dig: digitonin
EBV: Epstein–Barr virus
E–L: net electron transfer capacity (coupling efficiency)
E–P: net electron transfer reserve
E–R: respiratory reserve
fT4: free thyroxine
GpDH: glycerol-3-phosphate dehydrogenase
Gp/NS: fraction of NS-pathway attributable to GpDH
HIV: human immunodeficiency virus
MRI: magnetic resonance imaging
NS: combined NADH- and succinate-supported pathway
Omy: oligomycin
PBMCs: peripheral blood mononuclear cells
PM: pyruvate + malate
R: routine respiration
RCR: respiratory control ratio
ROX: residual oxygen consumption
Rot: rotenone
S: succinate
SARA: Scale for the Assessment and Rating of Ataxia
SUIT: substrate–uncoupler–inhibitor titration
TSH: thyroid-stimulating hormone
U: uncoupled electron transfer capacity
VZV: varicella-zoster virus
