# Supplementary material for: A Case Report of Reversible Mitochondrial Bioenergetic Dysfunction in PBMCs in Anti-GAD65–Associated Cerebellar Ataxia
Source: Cerebellum. 2026 May 20;25(3):83. doi: 10.1007/s12311-026-02025-y (PMC13186853; doi:10.1007/s12311-026-02025-y)
Supplement: Supplementary file 1 — Supplementary Material 1 [file 12311_2026_2025_MOESM1_ESM.docx]

**SUPPLEMENTARY MATERIAL**

**Material and Methods**

**Patient and control samples:**Peripheral blood (15 mL) was collected from a patient with anti-GAD–associated cerebellar ataxia at three time points: (1) before treatment, (2) after corticosteroid therapy, and (3) after plasmapheresis. A blood sample from an age- and sex-matched healthy individual served as the control. Samples were collected into EDTA tubes and processed within 30 minutes.

**PBMC isolation:**Peripheral blood mononuclear cells (PBMCs) were isolated from fresh EDTA-anticoagulated blood by Ficoll density gradient centrifugation using Histopaque as the separation medium. Isolated PBMCs were washed, counted, and resuspended in mitochondrial respiration medium (MiR05). Approximately 2 × 10⁶ cells were used per chamber for respirometric analysis.

**High-resolution respirometry:**Mitochondrial respiration was assessed using the Oroboros O2k high-resolution respirometry system (Oroboros Instruments, Innsbruck, Austria). All measurements were performed at 37°C in 2 mL chambers containing MiR05 medium. Oxygen consumption was recorded continuously, and respiratory states were normalized to maximal electron transfer (ET) capacity. Two complementary protocols were applied.

**SUIT protocol (Protocol 1):**A substrate–uncoupler–inhibitor titration (SUIT) protocol was used to quantify the individual contributions of Complex I (CI), Complex II (CII), and glycerol-3-phosphate dehydrogenase (GpDH) pathways. The sequence of substrates and inhibitors was as follows: pyruvate + malate (leak respiration and N-pathway entry), ADP (CI-linked OXPHOS), glutamate (enhanced CI-supported flux), succinate (combined CI+II, NS-pathway), cytochrome c (assessment of outer mitochondrial membrane integrity), rotenone (CI inhibition), glycerol-3-phosphate (GpDH-linked pathway), and antimycin A (residual oxygen consumption, ROX).

Parameters quantified included routine respiration, leak respiration, CI-linked OXPHOS, NS-pathway capacity, isolated CII capacity after rotenone, GpDH-supported respiration, and coupling indices (L/P, (P−L)/E, E−P).

**Intact PBMC CCP protocol (Protocol 2):**A second protocol was applied in intact PBMCs to evaluate overall mitochondrial efficiency, coupling, and bioenergetic reserve. The sequence of substrates and inhibitors was as follows: routine respiration (R), oligomycin (ATP synthase inhibition to assess proton leak), stepwise CCCP titration (maximal ET capacity, U), rotenone (CI inhibition), succinate (isolated CII-supported respiration), cytochrome c (membrane integrity test), digitonin (permeabilization control), and antimycin A (ROX).

Calculated parameters included L/R (relative leak), E−L (coupling efficiency), E−R (respiratory reserve), mitochondrial viability, and respiratory control ratio (RCR).

**
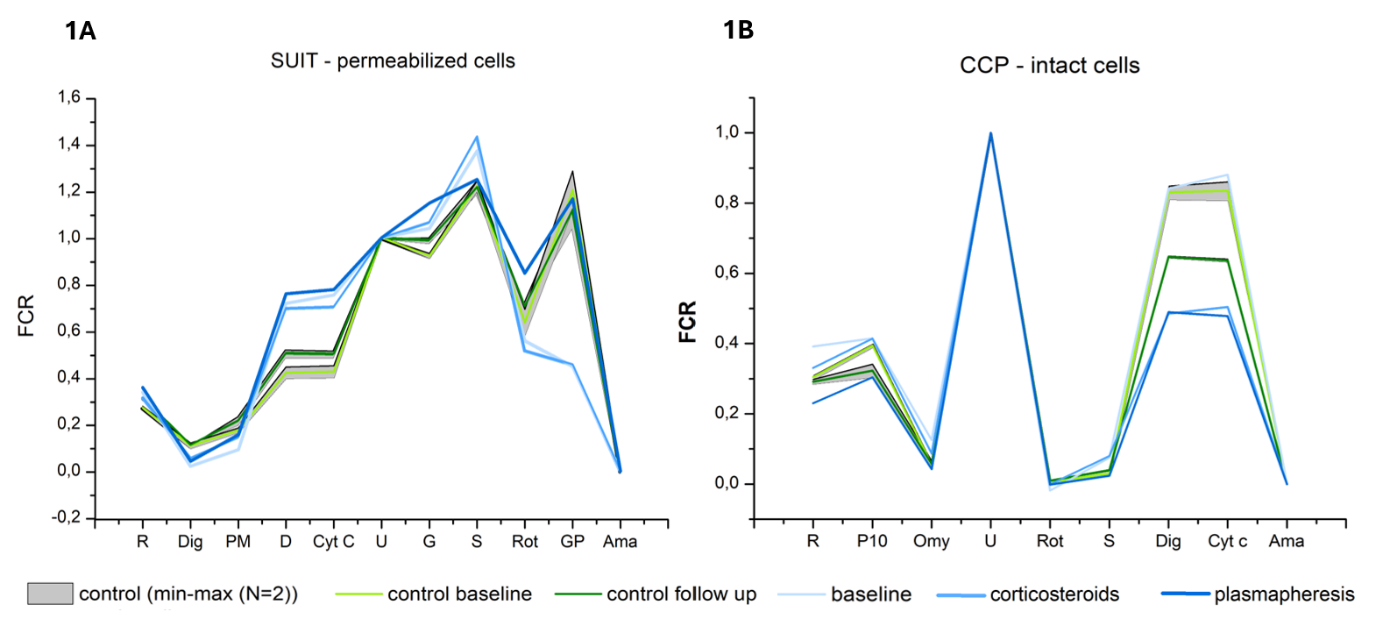
Figure 1A** **(1A)** SUIT protocol in permeabilized PBMCs illustrating the sequential engagement of Complex I, Complex II, and glycerol-3-phosphate–dependent respiration. At baseline and after corticosteroid therapy, respiration was characterized by reduced metabolic flexibility and limited reserve capacity. Following plasmapheresis, both intact and permeabilized cell profiles demonstrated increased maximal respiratory capacity and improved engagement of multiple respiratory pathways.

**(1B)** High-resolution respirometry profiles in intact and permeabilized PBMCs across treatment stages. **(A)** CCP protocol performed in intact peripheral blood mononuclear cells (PBMCs), demonstrating routine respiration (R), proton leak after oligomycin (Omy), maximal electron transport capacity (U), and residual oxygen consumption across control, baseline, post-corticosteroid, and post-plasmapheresis conditions.

**Normalization and Protein Quantification:**

Respiration rates were normalized to maximal electron transfer (ET) capacity (U = 1). Protein concentration in PBMC preparations was determined using a DC protein assay to enable standardized comparisons across samples. All respiratory states and coupling indices were compared across the three patient time points and against the healthy control. Data were processed according to standardized O2k analysis procedures.

**Methodological Consideration:**

Control PBMC samples were measured in duplicate using two independent O2k chambers. Respiration profiles were highly consistent across all respiratory states, indicating low technical variability of the respirometry measurements.

Due to limited patient material and confirmed technical reproducibility in control samples, the two O2k chambers were assigned to different protocols for patient measurements. One chamber was used for the CCP protocol in intact PBMCs, while the second chamber was used for the SUIT protocol in permeabilized PBMCs, allowing parallel assessment of mitochondrial function under both conditions.

Leak respiration was assessed differently in the two protocols. In permeabilized PBMCs, leak respiration represents NADH-linked proton leak measured in the presence of Complex I substrates. In intact PBMCs, leak respiration was defined as oligomycin-induced proton leak. As these measurements reflect distinct aspects of mitochondrial proton conductance, they are not directly comparable; however, together they provide complementary information on mitochondrial coupling under different experimental conditions.
